# Structured and Unstructured Physical Activity, Screen Time and Quality of Life in Childhood Cancer Survivors

**DOI:** 10.3390/cancers18050752

**Published:** 2026-02-26

**Authors:** Lauren Ha, Darcy Singleton, Claire E. Wakefield, Richard J. Cohn, David Simar, Christina Signorelli

**Affiliations:** 1 Behavioural Sciences Unit, Discipline of Paediatrics & Child Health, School of Clinical Medicine, Randwick Clinical Campus, UNSW Medicine & Health, UNSW Sydney, Sydney, NSW 2052, Australia; 2 Kids Cancer Centre, Sydney Children’s Hospital, Randwick, NSW 2031, Australia; 3 Division of Quality of Life and Pediatric Palliative Care, Stanford University, Stanford, CA 94305, USA; 4 Faculty of Medicine & Health, UNSW Sydney, Sydney, NSW 2052, Australia

**Keywords:** paediatric cancer, lifestyle behaviour, oncology, survivorship

## Abstract

**Background/Objectives:** Childhood cancer survivors face long-term cancer-related health risks, and daily behaviours, such as physical activity and recreational screen use, may influence their long-term overall wellbeing. Yet, little is known about survivors’ knowledge and understanding of recommended guidelines or their patterns of structured and unstructured activity. This study aimed to (i) assess survivors’ knowledge of physical activity and screen time guidelines, (ii) describe their self-reported (un)structured activity and screen use across weekdays and weekends, and (iii) examine associations with health-related quality of life. **Methods**: This secondary analysis used data from a feasibility pilot study of an intervention for survivors aged 8–13 years who had completed cancer treatment at least 12 months earlier. Participants self-reported physical activity, recreational screen use, knowledge of guidelines, and health-related quality of life (HRQoL). We used descriptive statistics and non-parametric tests to analyse behavioural patterns and associations. **Results:** Over one-third of survivors underestimated or overestimated the recommended physical activity and screen time (35%), whilst 23–50% reported ‘don’t know’. Participation in ‘Physical Education’ classes at school did not differ between summer and winter school terms, yet there was greater participation in structured physical activities in winter compared to summer school terms (median 270 vs. 170 min/week). For unstructured physical activities, 69% reported engaging in a median of 60 min/week at school on most days of the week in summer, whilst most survivors (96%) did not report any unstructured activity in winter. Only 19% met the recommended daily minimum of 60 min of physical activity year-round. Excessive screen use increased from school days (19%) to weekends (46%), notably handheld device screen time. We did not find any significant associations between meeting physical activity and screen time recommendations and HRQoL. **Conclusions**: Survivors frequently engage in irregular structured and unstructured physical activity levels and increased screen time. These findings emphasise the need for targeted, year-round interventions to improve physical activity, reduce recreational screen use, and support long-term survivorship health.

## Simple Summary

Many children who survive cancer face long-term health challenges, and daily habits like being active or spending time on screens can play an important role in their wellbeing. In this study, we explored how much physical activity and recreational screen time 8–13-year-old survivors report engaging in, and how aware they are of the recommended guidelines. We found that 85% and 58% of survivors were not aware of recommended limits for screen time and physical activity, 65% and 73% spent too much time on screens on weekdays and weekends, and 81% did not meet physical activity recommendations. Survivors who exceeded recommended screen time, or were less active, also reported poorer quality of life. These findings suggest that survivors need clearer guidance and support to build healthy lifestyles after cancer. By understanding these behaviours, researchers and clinicians can design better programmes that help survivors stay active, limit screen use, and improve their long-term health after cancer.

## 1. Introduction

As childhood cancer survival rates have improved in recent decades, so too has awareness of the associated long-term treatment-related morbidities [1,2]. Long-term health problems may present diversely as physical, cognitive, or psychosocial sequelae, which can emerge years after treatment [2]. Cardiovascular disease is the leading non-cancer cause of premature mortality among childhood cancer survivors [3], and causes the most life years lost among survivors [4]. Other persistent physical health challenges include obesity, reduced pulmonary or endocrine function, and neurocognitive impairment [5]. Evidence consistently demonstrates that regular physical activity reduces the risk of such late effects, yet survivors engage in substantially less physical activity—up to 70% less—than their peers without cancer, and the majority (85%) do not meet the recommended Australian or World Health Organization (WHO) guidelines [6,7,8].

Physical activity has been recognised internationally as safe and beneficial for any child and adolescent diagnosed with cancer [9,10]. Yet, multiple physical (fatigue, weakness, pain) and psychosocial barriers (motivation, appearance concerns, fear of injury, or perceived difference from peers) may hinder survivors’ participation [11,12,13]. Physical activity participation also varies between structured (e.g., planned, organised sports, e.g., soccer, swimming) and unstructured (e.g., free play, outdoor games, non-organised activity) forms, and both play an essential role in children’s growth, development and wellbeing. Most research to date has predominantly focused on patient and survivor engagement in structured physical activity, overshadowing other aspects of physical activity including unstructured forms such as playing during school lunch times. Previous studies in children without cancer report that they accumulate most of their physical activity during lunch times and school [14,15]. Characterising these patterns in childhood cancer survivors may further identify and support implementation strategies to promote long-term physical activity behaviours, informing future multifaceted interventions that are developmentally appropriate, cost-effective, and sustainable.

Alongside insufficient activity, excessive sedentary behaviour including recreational screen use poses additional health risks. Prolonged screen exposure in youth has been linked with increased body mass index, waist circumference, and blood pressure, all of which contribute to poorer cardiometabolic outcomes [16,17]. Guidelines globally recommend limiting recreational screen time to under two hours per day [9,18,19]. Yet, screen time in children without cancer has increased in recent years, and limited data in childhood cancer survivors suggest two-thirds exceed these recommended limits [20]. Furthermore, device-specific screen use has been shown to differ among Australian children without cancer. Passive screen time (e.g., television [TV]) has been linked to poorer psychological and educational outcomes, whereas educational screen time showed improvements in educational outcomes [21]. To date, screen time research has not differentiated by device type (e.g., TV, smartphone/tablet, computer) among childhood cancer survivors. Taken together, inadequate physical activity and excessive screen time can result in significantly reduced health-related quality of life (HRQoL) in young survivors [22,23]. Encouraging positive lifestyle behaviours and strengthening survivors’ knowledge of recommendations may support survivors’ HRQoL and empower them to adopt healthier behaviours to mitigate the long-term impact of cancer.

This study aims to better characterise screen time and physical activity behaviours in childhood cancer survivors, specifically by (i) understanding survivors’ awareness of screen time and physical activity recommendations, (ii) describing survivors’ self-reported screen-based behaviours and structured and unstructured physical activity levels across a typical week (weekdays and weekends), and (iii) assessing the association between screen use, physical activity, and HRQoL. These data will provide necessary evidence to inform the design and implementation of survivorship interventions to promote active lifestyles and reduce sedentary behaviour among survivors of childhood cancer.

## 2. Materials and Methods

This was a secondary analysis from a single-centre cross-sectional pilot study of a physical activity intervention called ‘iBounce’, published elsewhere [24]. The study was approved by the Sydney Children’s Hospital Network Human Research Ethics Committee (HREC/18/SCHN/471).

### 2.1. Participants

Survivors were eligible if they (i) were aged between 8 and 13 years, (ii) were diagnosed with cancer as a child (<16 years), (iii) were at least 12 months post cancer treatment completion or were undergoing maintenance chemotherapy, (iv) were able to communicate in English, and (v) had internet access at home. Survivors were excluded if they (i) had a post-recruitment cancer relapse at the time of participation, (ii) had a medical condition that would prohibit exercise, or (iii) had participated in another intervention research study less than 4 weeks prior.

We recruited eligible survivors between May 2019 and May 2021 from Sydney Children’s Hospital, Australia. Nursing staff identified eligible participants through hospital clinic lists, and the study coordinator contacted the parents of those approved by treating oncologists.

### 2.2. Data Collection

The primary outcome measures for this study were self-reported physical activity levels. Secondary outcomes included screen-based behaviours and knowledge of the recommended physical activity and screen time limits. Parents reported their child’s clinical and demographic factors including diagnosis group, age, gender, home postcode (i.e., rural status), annual household income and highest level of educational attainment of parents.

#### 2.2.1. Physical Activity

Our questionnaire was adapted from the validated New South Wales (NSW) government ‘School Physical Activity and Nutrition Survey’ (SPANS) [25,26]. Children self-reported their physical activity levels across the Australian school terms in summer (Terms 1 and 4) and winter (Terms 2 and 3). Structured physical activity, such as school-based physical education and extra-curricular team sports, and unstructured physical activity were self-reported in terms of frequency (times per week) and duration (minutes per week). Additionally, survivors self-reported the number of days over the past week they engaged in >60 min of moderate–vigorous physical activity (MVPA), defined as *“any activity that increases your heart rate and gets you out of breath”*.

#### 2.2.2. Recreational Screen Time

Survivors self-reported their recreational (i.e., non-educational) screen time during weekdays and weekends for computer or gaming console usage, handheld devices usage (i.e., iPad, tablet, mobile phone), and television usage. Survivors self-reported the frequency (i.e., number of times per week) and duration of their screen time behaviours (‘no time’, ‘1–30 min’, ‘30–60 min’, ‘1–2 h’, ‘2–3 h’, ‘3–4 h’, ‘4–5 h’ and ‘5+ h’).

#### 2.2.3. Knowledge of Physical Activity and Screen Time Limits

Knowledge of the relevant physical activity and screen time limits were assessed by asking survivors to self-report the number of minutes of physical activity and screen time recommended for school-aged children per day, including the option of ‘don’t know’.

#### 2.2.4. Health-Related Quality of Life

Survivors’ HRQoL was assessed using the EQ-5D Youth Questionnaire (EQ-5D-Y-5L) [27]. The EQ-5D-Y-5L includes five dimensions of quality of life: mobility, looking after oneself, doing usual activities, pain or discomfort, and feeling worried, sad or unhappy. Participants answered on a 5-point Likert scale (1 = no problems to 5 = I am unable to), with lower scores indicating better quality of life. This questionnaire has been validated in childhood cancer survivors (test–retest reliability of 0.84) [28].

### 2.3. Statistical Analysis

We used IBM SPSS Statistics 28.0 (IBM Corp.) for descriptive statistics and R (version 2024.09.0) for statistical analyses. To assess awareness of recommended physical activity and screen time limits, we compared survivors’ answers to the relevant WHO and Australian government guidelines, which recommend at least 1 h of physical activity and a maximum of 2 h of non-educational screen time daily [9,29]. To determine whether survivors’ awareness of limits differed by parental education, we dichotomized survivor’s knowledge to ‘aware’ (i.e., correctly identified recommendations) and ‘not aware’ (incorrectly identified recommendations, including those who reported ‘don’t know’) and used Fisher’s exact test, including reporting odds ratios (ORs) with 95% confidence intervals (CIs) to indicate strength and direction of associations. To assess differences in recreational screen time distribution between weekdays and weekends for each category (computer, handheld device, television), we used the Wilcoxon signed-rank test. We dichotomized screen time as ≤2 h (i.e., meeting the recommended guidelines) and >2 h (exceeding the recommended guidelines). To evaluate the difference in screen time between weekday and weekend, we used McNemar’s test. To assess the association between meeting screen time and physical activity recommended guidelines, we used Fisher’s exact test. To assess the association between HRQoL and physical activity or screen time, we used Wilcoxon signed-rank test. Statistical significance was set at *p* < 0.05 (two-tailed).

## 3. Results

Of 72 invited survivors, 30 opted into the study and 27 completed baseline questionnaires. One participant had incomplete survey responses in the variables used in this secondary analysis; therefore, we included 26 participants in this study. Participating survivors’ mean age was 10.1 ± 1.4 years, 46% were female, 85% were living in a major city, 54% of parents had a university or postgraduate degree (46% of parents completed high school, apprenticeship, certificate or diploma) and 46% of parents earned $90,000 AUD or more of annual income (23% of parents did not provide a response). Over half of survivors were diagnosed with Acute Lymphoblastic Leukaemia (ALL; n = 15) and the mean time since treatment completion was 5.0 ± 3.1 years.

### 3.1. Awareness of Physical Activity and Screen Time Limits

Nine survivors (35%) incorrectly reported the physical activity recommendations. Of those who were incorrect, five survivors overestimated and four survivors underestimated the limits. Six survivors (23%) reported that they ‘don’t know’. Of the 15 survivors (58%) who were not aware of the physical activity recommendations, seven had parents with higher education. Parent education was not associated with survivors’ awareness of physical activity guidelines (OR 0.51, 95% CI [0.07–3.13], *p* = 0.453).

Nine survivors (35%) incorrectly reported screen time limits. Thirteen survivors (50%) reported ‘don’t know’. Of the four survivors (15%) who correctly reported screen time limits, one survivor self-reported meeting those guidelines while three survivors self-reported 5 or more hours of screen time on weekdays or weekends. Of the 22 survivors (85%) who were not aware of the screen time limits, 11 had parents with higher education. Parent education was not associated with survivors’ awareness of screen time limits (OR 0.35, 95% CI [0.005–5.12], *p* = 0.598).

Four survivors (15%) who met screen time guidelines also met physical activity guidelines, compared to 54% (n = 14) who did not meet either guideline. Meeting screen time guidelines was not significantly associated with meeting physical activity guidelines (OR 3.53, 95% CI [0.43–33.71], *p* = 0.188).

### 3.2. Engagement in Physical Activity Levels (Structured and Unstructured)

Participation in Physical Education (PE) at school did not significantly differ between summer and winter school terms for frequency (V = 10, *p* = 0.571) or duration per week (V = 6, *p* = 0.786, Table 1).

In summer school terms, six (23%) survivors met recommended guidelines and in winter school terms, eight (31%) met recommended guidelines. Five survivors (19%) maintained recommended physical activity levels in both summer and winter school terms.

There were significantly more sports played in winter compared to summer school terms (V = 15, *p* = 0.003). The median total minutes of sports played per week were lower in summer (median = 195) compared to winter (median = 270), indicating greater participation in the winter school terms (V = 58.5, *p* = 0.048).

In summer school terms, 69% of survivors (18/26) reported engaging in a median of 60 min per week of unstructured physical activity during recess and lunch at school on most days of the week (median 4.5 days/week for recess and 4 days/week for lunch; Table 2). Outside of school, 48% (12/26) reported physical activity on median 2.5 days/week and median 120 min/week. Only one participant reported unstructured physical activity during winter school terms. There were no reported outside-of-school activities in winter.

### 3.3. Engagement in Recreational Screen Time

**Figure 1:**
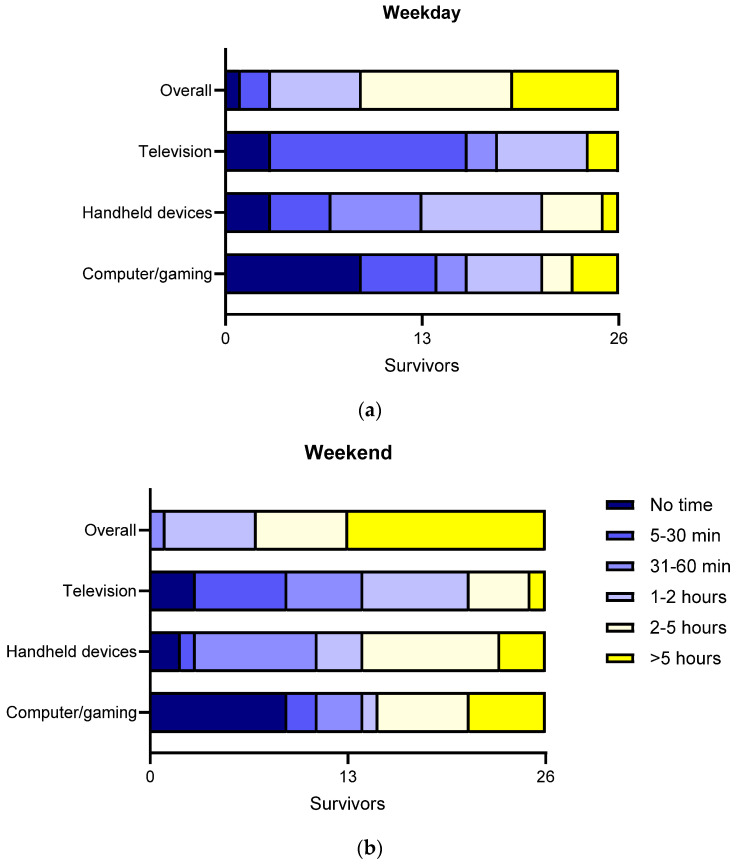
Distribution of recreational screen time on a (**a**) weekdays and (**b**) weekends, by type of device. Bars in yellow (i.e., ‘2–5 h’ and ‘>5 h’ categories) denote exceeding recommended recreational screen time guidelines.

The proportion of survivors meeting overall screen time recommended guidelines was 35% (9/26) on weekdays and 27% (7/26) on weekends (Figure 1). Of those that did meet screen time recommendations, only one survivor correctly reported the recommendations, whilst six reported ‘don’t know’. Survivors reported spending 5–30 min watching TV per day (13/26), 1–2 h on their handheld devices (8/26) and spending no time on their computer or gaming console (9/26) on weekdays (Figure 1). On weekends, survivors reported spending 1–2 h watching TV per day (7/26), 2–5 h on their handheld device (9/26), and no time on the computer or gaming console (9/26).

Handheld device (i.e., iPad, tablet or phone) screen time was significantly higher on weekends compared to weekdays (V = 0, *p* = 0.003). The proportion of survivors who exceeded the recommended 2 h of handheld device time significantly increased from 19% on weekdays to 46% on weekends (χ^2^ = 4.17, df = 1, *p* = 0.04).

There were no significant differences between weekdays and weekends for overall screen time (V = 0, *p* = 0.37), computer/gaming (V = 4, *p* = 0.11) and television screen time (V = 9, *p* = 0.44). The proportion of survivors who exceeded 2 h of computer/gaming (17% vs. 35%, χ^2^ = 3.2, df = 1, *p* = 0.07) and television (8% vs. 19%, χ^2^ = 0.5, df = 1, *p* = 0.48) screen time was not statistically different on weekdays compared to weekends.

### 3.4. Association with Quality of Life

Survivor-reported HRQoL is reported in Table 3. Most survivors reported ‘no problems’ in self-care (100%), mobility (73%), and usual activities (73%).

The median total HRQoL score was higher among survivors who did not meet screen time recommendations (median = 7, n = 17) compared with those who did meet recommendations (median = 5, n = 9), although this was not significantly different between groups (W = 104, *p* = 0.122).

The median total HRQoL score was also higher among survivors who did not meet physical activity recommendations (median = 7, n = 19) compared with those did not meet recommendations (median = 6, n = 7), although this was not significantly different between groups (W = 84, *p* = 0.305).

## 4. Discussion

This study suggests that many childhood cancer survivors engage in low levels of structured and unstructured physical activity, have high screen time and do not know what the recommended limits of physical activity or screen time guidelines are. Extending prior research, our findings demonstrate that these suboptimal behaviours co-occur among a cohort of young long-term survivors. These results highlight the need for targeted interventions to foster healthier behaviours aimed at optimising quality of life.

A large proportion of survivors in our study incorrectly reported WHO guidelines for screen time and physical activity, suggesting significant gaps in survivor health literacy that may limit engagement in recommended health behaviours. It is critical to empower survivors, and caregivers of young patients, with knowledge about their cancer history and resulting risk of late effects, to enable them to make more active health and lifestyle choices [30]. Studies internationally show that survivors report not receiving such information, compounded by suboptimal guideline knowledge and thus engagement with the recommendations [30,31]. Further complicating the delivery and retention of information is families’ differing preferences as well as the challenge of ensuring that information delivered to caregivers of very young survivors is passed on when they are of age [32]. This suggests the need for targeted education to improve survivor and family understanding of these fundamental lifestyle recommendations.

A significant proportion of survivors in our study reported not achieving the WHO guideline of 60 min of MVPA daily (77% during summer terms and 69% during winter terms), and only 19% sustained adequate activity levels across both summer and winter school terms. The high prevalence of insufficient physical activity observed in our cohort aligns with findings from other large survivor cohorts, suggesting that this is an international challenge [33,34,35]. These findings underscore survivors’ elevated risk of inactivity and the urgency for structured support systems and school-based opportunities to facilitate year-round physical activity. Although many children who were active at school engaged in physical activity on most days, nearly one-third (31%) in our sample reported no school-based activity at all, highlighting a key opportunity for schools to promote movement. Further, participation in structured physical activity was greater in winter compared to summer school terms, which may reflect patterns specific to the Australian context. Given that most participants were around 10 years old, encouraging activity at this age is essential to support the development of long-term healthy habits. In our study, unstructured physical activity also played a key role in survivors’ movement patterns during school (69%) and outside of school (48%). This suggests that free play and games may offer an important and often overlooked avenue to engage highly sedentary survivors in enjoyable, sustainable activity, and are potentially more motivating than highly structured programmes [36]. However, additional support may be required for some survivors, including those with difficulties with social skills, to facilitate confidence, inclusion and successful participation in less structured contexts [37].

More survivors exceeded screen time on the weekends compared to school days. These findings suggest that excessive screen time may be more problematic on days without structured routines, potentially compounding sedentary habits and contributing to adverse health outcomes. This pattern warrants attention from families and healthcare teams and may benefit from tailored interventions during weekends. Many survivors in our sample did not meet recommended screen time limits (35% weekdays; 27% weekends), notably lower than population estimates showing that 62% of Australian children of a similar age met weekday screen time recommendations and 21% on weekends [25,26]. This disparity highlights that survivors may be at heightened risk for excessive screen use compared to their peers, particularly mobile device use, warranting targeted education or interventions to address screen-based behaviours.

In our sample, more than half (54%) of survivors failed to meet either screen time or physical activity guidelines, and both excessive recreational screen time and insufficient activity were not significantly associated with HRQoL. Yet, a recent Cochrane review on physical activity interventions suggests clear psychosocial benefits for survivors in terms of improved wellbeing and quality of life, highlighting it as critical component of cancer survivorship care [38]. Screen time and physical activity guideline achievement were also not significantly correlated in our study, as observed in other survivor cohorts [39]. Whilst reduced screen time may mean more opportunity for physical activity, analyses in other survivors has similarly highlighted that reductions in screen time do not necessarily automatically translate to increased physical activity, and vice versa [40]. This aligns with evidence from a recent dose–response analysis in school-aged children, which found that meeting both physical activity and screen time recommendations (rather than just one) was associated with the most favourable mental health outcomes, further underlining the importance of independent consideration and intervention for both behaviours [41].

Our findings should be interpreted in light of key limitations, including the small sample size, low response rate and recruitment from a single metropolitan centre, resulting in low representation of rural and remote families within NSW, Australia. Participation was also limited to English-speaking families, which may limit generalisability to culturally and linguistically diverse populations. Additionally, non-recreational (e.g., educational) screen time was not assessed in our study, which is an important contributor to overall screen time in this population. This study relied on self-report, which may not represent accurate measures of survivors’ physical activity levels and screen time. We also excluded objective physical activity measurements from this study due to low adherence to activity trackers, consistent with challenges reported in our original study [24]. Finally, we did not assess parents’ awareness of physical activity or screen time limits, which may influence survivors’ knowledge and behaviours. Future research should consider parent factors given the age of this population.

## 5. Conclusions

These data highlight that most childhood cancer survivors in our study have limited knowledge of physical activity and screen time limits, which may contribute to insufficient physical activity and excessive recreational screen use. These suboptimal lifestyle behaviours are linked to poorer quality of life, underscoring the need for targeted education and interventions that address both physical activity engagement and screen time reduction. Tailored, year-round support in schools and families is essential to promote sustained healthy behaviours and improve long-term survivorship outcomes that mitigate the impact of cancer.

## Figures and Tables

**Table 1:** Structured physical activity levels (i.e., planned, organised sports) among 26 survivors.

Structured Physical Activity	Summer Terms Median (IQR)/n (%)	Winter Terms Median (IQR)/n (%)	*p*-Value *^+^*
Physical Education (PE)			
Times per week	1 (1–2)	1.5 (1–2)	0.571
Minutes per week	60 (60–120)	60 (60–120)	0.786
Number of sports played ^1^			**0** **.003**
0	7 (26%)	4 (15%)	
1	14 (54%)	10 (38%)	
2	4 (15%)	9 (35%)	
3	1 (4%)	1 (4%)	
4	0 (0%)	2 (8%)	
Sports minutes per week	195 (120–330)	270 (180–492.5)	**0** **.048**

^+^ Wilcoxon signed-rank test. ^1^ Sport types in summer: n = 6 swimming, n = 5 football, n = 3 basketball, n = 2 tennis, n = 2 cricket, n = 1 netball, n = 2 martial arts, n = 1 hockey, n = 1 athletics, n = 1 nippers (i.e., surf skills and safety), n = 1 Australian Oztag, n = 1 dance. Sport types in winter: n = 12 football, n = 7 swimming, n = 4 netball, n = 3 basketball, n = 3 tennis, n = 2 boxing, n = 2 hockey, n = 2 dance, n = 2 Australian Oztag, n = 1 nippers, n = 1 martial arts. NB: *p*-values in bold indicate statistical significance (*p* < 0.05).

**Table 2:** Unstructured physical activity levels (i.e., casual play) among 26 survivors.

Unstructured Physical Activity	Summer Terms Median (IQR)/n (%)	Winter Terms n (%)
During recess ^1^	18 (69%)	1 (4%)
Times per week	4.5 (1–5)	2
Minutes per week	60 (56–97)	60
During lunch ^2^	18 (69%)	1 (4%)
Times per week	4 (2–5)	1
Minutes per week	60 (56–195)	30
Outside of school ^3^	12 (48%)	0 (0%)
Times per week	2.5 (1.75–5.5)	-
Minutes per week	120 (60–180)	-

^1^ Recess activity types in summer: n = 7 running around/games, n = 3 handball, n = 3 football, n = 2 walking, n = 2 tag, n = 1 traditional indigenous games. Recess activity types in winter: n = 1 climbing wall. ^2^ Lunch activity types in summer: n = 6 running around/games, n = 4 handball, n = 2 football, n = 2 group games, n = 1 beach swimming, netball n = 1, n = 1 skipping, n = 1 trampoline. Lunch activity types in winter: n = 1 cycling. ^3^ Outside-of-school activities in summer: n = 4 walking/walking the dog, n = 3 football, n = 2 bike riding, n = 1 swimming, n = 1 miscellaneous activities. There were no reported outside-of-school activities in winter.

**Table 3:** EQ-5D-5L frequencies and proportions reported by dimension and level.

	Mobility n (%)	Self-Care n (%)	Usual Activities n (%)	Pain/Discomfort n (%)	Anxiety/Depression n (%)
No problems	19 (73%)	26 (100%)	19 (73%)	12 (46%)	17 (65%)
Slight problems	7 (27%)	0 (0%)	4 (15%)	14 (54%)	6 (23%)
Moderate problems	0 (0%)	0 (0%)	2 (8%)	0 (0%)	2 (8%)
Severe problems	0 (0%)	0 (0%)	0 (0%)	0 (0%)	1 (4%)
Extreme problems/unable to do	0 (0%)	0 (0%)	1 (4%)	0 (0%)	0 (0%)

## Data Availability

The data presented in this study are available on request from the corresponding author due to privacy reasons.

## Abbreviations

The following abbreviations are used in this manuscript:

CVD	Cardiovascular Disease
HRQoL	Health-Related Quality of Life
HREC	Human Research Ethics Committee
MVPA	Moderate-to-Vigorous Physical Activity
NSW	New South Wales
SPANS	School Physical Activity and Nutrition Survey
WHO	World Health Organization
P.E.	Physical Education
OR	Odds Ratio
CI	Confidence Interval
EQ-5D-Y-5L	EuroQol 5-Dimension Youth Questionnaire 5-Level
PA	Physical Activity
